# Ultrasound modulates microglial activity and reduces neuroinflammation in a parameter-dependent manner

**DOI:** 10.1038/s44384-026-00047-8

**Published:** 2026-05-06

**Authors:** Sarina Grewal, Francesco Iacoponi, Lok Yin Nicholas Chan, Valeria Dosso, William Lim Kee Chang, Vanessa Drevenakova, Albert Ugwudike, Leonardo Ricotti, Paul M. Matthews, Andrea Cafarelli, Sophie V. Morse

**Affiliations:** 1 Department of Bioengineering, Imperial College London, London, UK; 2 Department of Brain Sciences, Imperial College London, London, UK; 3 UK Dementia Research Institute, Imperial College London, London, UK; 4 The BioRobotics Institute, Scuola Superiore Sant’Anna, Pisa, Italy; 5 Department of Excellence in Robotics & AI, Scuola Superiore Sant’Anna, Pisa, Italy; 6 Department of Chemistry, Imperial College London, Molecular Science Research Hub, White City, London, UK; 7 Department of Computing, Imperial College London, London, UK; 8 Rosalind Franklin Institute, Harwell Science and Innovation Campus, Didcot, Oxon UK

**Keywords:** Diseases, Immunology, Neurology, Neuroscience

## Abstract

Neuroinflammation contributes to the progression of many neurological diseases. Here, we explore whether ultrasound can reduce microglia-mediated inflammation in vitro and in vivo. We tested a broad range of ultrasound parameters in a BV2 microglial cell line, treated with lipopolysaccharide (LPS) to induce an inflammatory response. We found that specific combinations of centre frequency, acoustic pressure and treatment duration can significantly lower the levels of pro-inflammatory cytokines, including tumor necrosis factor (TNF)-α, interleukin (IL)-1β and IL-6. These effects lasted up to 72 h and were associated with the downregulation of the nuclear factor κB (NF-κB), suggesting a mechanistic link between ultrasound and inflammation. Further investigation in vivo, in LPS-treated mice, revealed a reduction in TNF-α expression in the hippocampus following ultrasound. Overall, our findings showcase the potential of ultrasound as a non-invasive therapeutic strategy to reduce neuroinflammation and restore brain homeostasis.

## Introduction

Neuroinflammation can drive the pathology of several neurological diseases, including Alzheimer’s disease, Parkinson’s disease, ischemic stroke and traumatic brain injury^1^. Although inflammation is initially protective and temporary, a sustained inflammatory response can disrupt brain homeostasis and contribute to disease progression^2^.

Microglia are the brain’s resident immune cells and play an integral role in mediating inflammation in the central nervous system^3^. They exert a multitude of physiological functions, including synaptic plasticity, clearance of cellular debris through phagocytosis and immune surveillance^4–6^. Notably, microglia are highly dynamic cells and continuously monitor their microenvironment by extending and retracting their processes^7,8^. In response to brain damage or injury, they alter their morphology and rapidly trigger an immune response by secreting signalling molecules such as chemokines and cytokines that aim to contain the damage^9^. However, a continuous release of NF-κB-regulated pro-inflammatory cytokines, such as TNF-α, IL-1β and IL-6, can amplify inflammation and exacerbate pathology^10^. Therefore, it is of prime significance to develop therapeutic interventions to modulate microglia-mediated inflammation.

Given the significant challenges of developing safe and effective treatments for neurological diseases, novel approaches are being investigated^11,12^. Ultrasound is a non-invasive technology that can treat brain tissue without relying on pharmacological agents^13^. Ultrasound waves induce mechanical effects that can result in changes to cellular membranes, including the activation of mechanosensitive ion channels such as PIEZO1^13^. Several animal and human studies have shown that ultrasound can cause an influx of intracellular calcium in neurons and induce changes in membrane potential, leading to inhibitory or excitatory effects^14–16^. Emerging evidence shows that ultrasound can also influence microglial functions and inflammatory responses in disease^17–19^.

Microglia are known to respond to mechanical cues and have been shown to migrate to sites of inflammation following ultrasound exposure (centre frequency = *f*: 2.25 MHz, intensity = *I*: 0.31 to 1.16 mW/cm², treatment duration = *t*: 20 s)^20^. Increases in the cell density of microglia stained positively for ionized calcium-binding adaptor molecule 1 (Iba1) has been reported in ultrasound-treated brain regions in mice (*f*: 0.6 MHz, pressure: 260 to 270 kPa, *t*: 95 s)^21^. In Alzheimer’s pathology, enhanced microglial phagocytosis of amyloid plaques was observed following ultrasound treatment (*f*: 1 MHz, *I*: 294 mW/cm², *t*: 30 min), with an increase in anti-inflammatory IL-10 protein expression in the hippocampus of APP/PS1 mice^22^. These results suggest that ultrasound can modulate microglial activity to promote their protective functions in disease. Additionally, in Parkinson’s disease, ultrasound (*f*: 1 MHz, *I*: 528 mW/cm², *t*: 15 min) treatment has been shown to reduce IL-1β levels and enhance glial cell-line-derived neurotrophic factors, which promote the survival of dopaminergic neurons in the substantia nigra^23^. Following ultrasound (*f*: 1 MHz, *I*: 30 mW/cm², *t*: 15 min) in vitro, microglial cells treated with LPS to induce inflammation have been shown to promote the release of anti-inflammatory cytokine arginase-1, which is involved in tissue repair and the resolution of inflammation^24^. Despite these promising findings, little is known about how varying ultrasound parameters can shape microglial inflammatory responses.

In this study, we investigated how varying ultrasound centre frequencies (0.5, 1, 2 MHz), acoustic pressures (0.1, 0.2, 0.4 MPa) and treatment durations (5, 30, 60 min) alter microglial inflammation. Given the diverse ultrasound parameters reported in previous microglial and neuroinflammation studies, we selected a broad range of parameters from low to high^18–20,22,23^. This allows us to systematically map how each parameter and its combinations influence microglial responses under inflammatory conditions. We identified parameters that significantly attenuated microglia-mediated inflammation in vitro and subsequently validated the findings in a neuroinflammation mouse model. Our results reveal a parameter-dependent modulation of microglial activity with ultrasound, which provides critical insights into how this technology could be harnessed to modulate immune responses in neurological diseases for advanced therapies.

## Results

### Ultrasound centre frequency differentially modulates inflammation in microglia

**Fig. 1:**
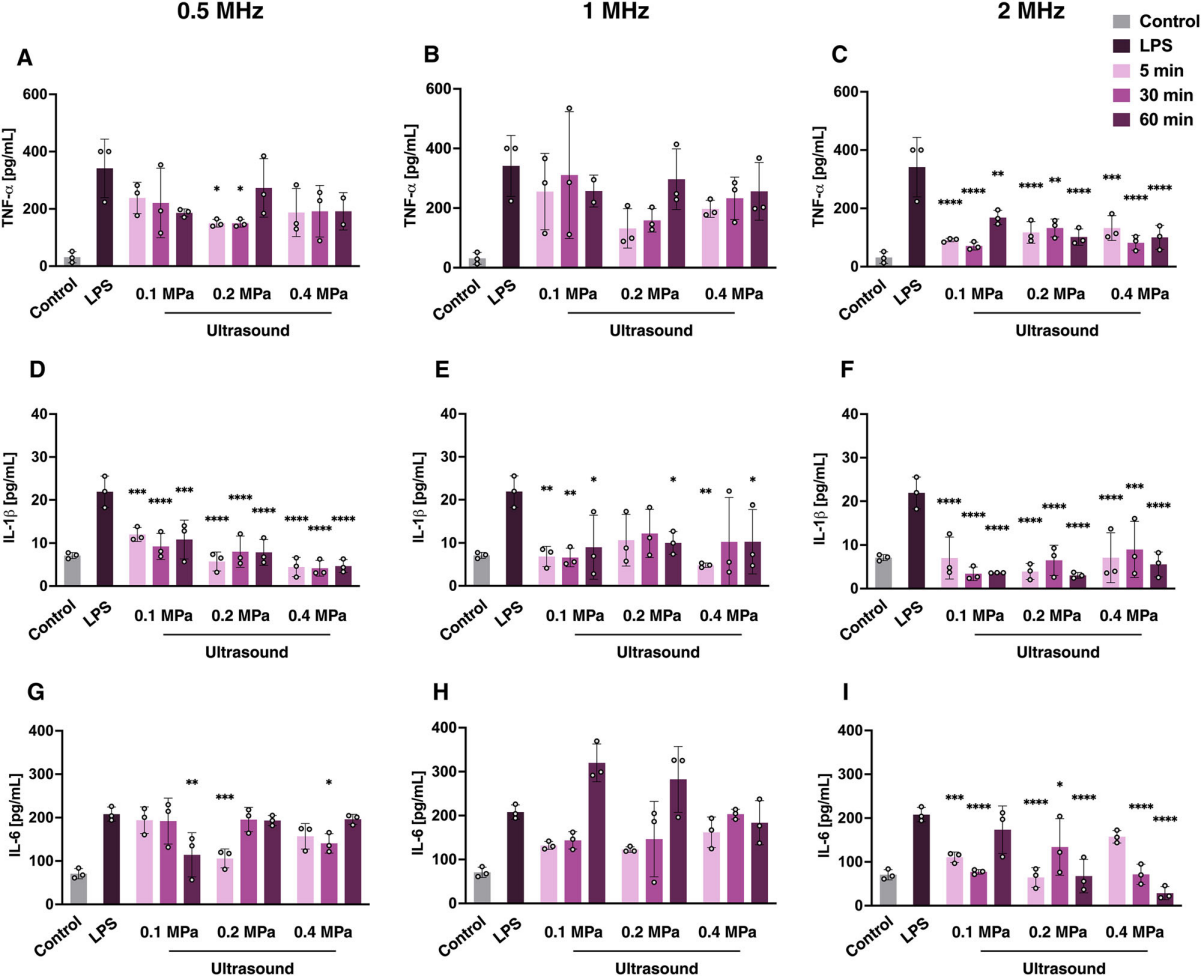
Ultrasound centre frequency differentially modulates inflammation in microglia. Quantification of TNF-α, IL-1β and IL-6 levels in LPS-treated microglia is shown following ultrasound treatment at different frequencies (0.5, 1, 2 MHz), acoustic pressures (0.1, 0.2, 0.4 MPa) and durations (5, 30, 60 min). Cytokine concentrations were measured by ELISA 24 h after ultrasound (**A**–**I**). Data is shown as means ± SD (*n* = 3). Statistical analysis was performed by two-way analysis of variance (ANOVA) with Tukey’s post hoc test relative to LPS-treated controls; *p < 0.05, **p < 0.01, ***p < 0.001, ****p < 0.0001.

First, we explored how varying ultrasound centre frequency, acoustic pressure, and treatment duration affected the release of pro-inflammatory cytokines in LPS-treated microglial cells (Fig. 1). Overall, the strongest reductions in cytokine levels were observed when ultrasound was applied at 0.5 and 2 MHz, while increasing acoustic pressure or treatment duration did not produce consistent trends in cytokine reduction across the parameters investigated.

LPS treatment led to significantly higher levels of all cytokines compared to the untreated controls (Fig. 1). In terms of TNF-α levels, at 0.5 MHz, ultrasound reduced LPS-elevated TNF-α levels, with significant decreases at 0.2 MPa following 5 and 30 min of exposure (*p* < 0.05; Fig. 1A). In contrast, ultrasound at 1 MHz did not produce significant changes in TNF-α across the acoustic pressures or durations tested (Fig. 1B). The most pronounced suppression of TNF-α release was achieved at 2 MHz, which significantly reduced TNF-α concentrations at all pressures and durations compared with LPS-treated controls (*p* < 0.001 to *p* < 0.0001; Fig. 1C).

When analysing IL-1β levels, the strongest reductions relative to the elevated LPS-induced levels were observed at 0.5 and 2 MHz across all tested pressures and treatment durations (Fig. 1D–F). At 1 MHz, IL-1β levels were significantly reduced at 0.1 MPa across all durations, with additional decreases at 0.2 MPa after 60 min (*p* < 0.05) and at 0.4 MPa after 5 min (*p* < 0.01) and 60 min (*p* < 0.05) of treatment (Fig. 1E).

IL-6 levels were also significantly reduced under several ultrasound conditions. At 0.5 MHz, IL-6 decreased at 0.1 MPa after 60 min of treatment (*p* < 0.01), at 0.2 MPa after 5 min (*p* < 0.001), and at 0.4 MPa after 30 min of treatment (*p* < 0.05; Fig. 1G). In contrast, no significant changes were observed at 1 MHz across the tested pressures (Fig. 1H). At 2 MHz, IL-6 levels were markedly reduced at all pressures compared with LPS, with effects observed at 0.1 MPa after 5 (*p* < 0.001) and 30 min (*p* < 0.0001), at 0.2 MPa across all durations (*p* < 0.05 to *p* < 0.0001), and at 0.4 MPa after 30 and 60 min of ultrasound (*p* < 0.0001; Fig. 1I).

### Minimal thermal effects across all ultrasound parameters

**Fig. 2:**
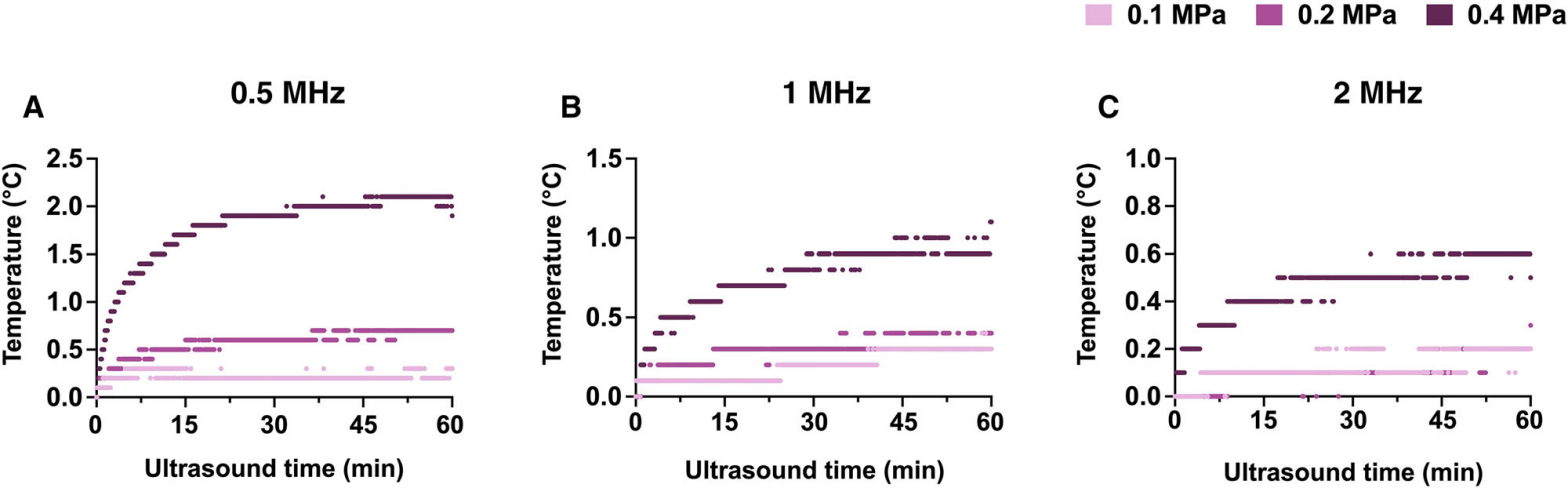
Temperature measurements show minimal thermal effects across all ultrasound parameters. Temperature rises (°C) were recorded with a thermocouple probe during ultrasound treatment at different frequencies: 0.5 MHz (**A**), 1 MHz (**B**), and 2 MHz (**C**); and acoustic pressures (0.1, 0.2, 0.4 MPa) for up to 60 min (**A**–**C**).

To investigate the potential contribution of thermal effects, we measured the temperature within the device across all parameter combinations (Fig. 2). In alignment with the International Transcranial Ultrasonic Stimulation Safety and Standards (ITRUSST) guidelines, which consider thermal risks negligible when temperature rises remain below 2 °C, our results indicate that nearly all ultrasound parameter combinations in this study produced minimal thermal effects^25,26^. At the higher frequencies of 1 and 2 MHz, temperature increases remained below 2 °C across all pressures and treatment durations. A temperature rise of 2.1 °C was observed only at 0.5 MHz, at the highest acoustic pressure (0.4 MPa) following 60 min of ultrasound.

### Ultrasound reduces cytotoxicity in microglia

**Fig. 3:**
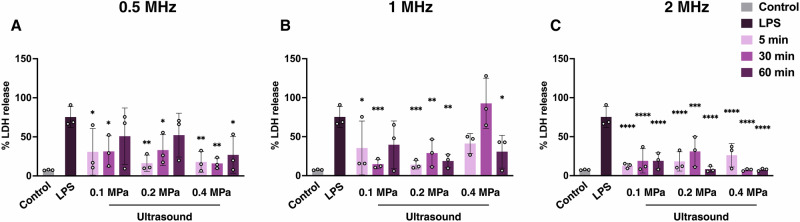
Ultrasound reduces cytotoxic responses in microglia. LDH release was quantified in LPS-treated microglia 24 h after ultrasound treatment at varying frequencies (0.5, 1, 2 MHz), acoustic pressures (0.1, 0.2, 0.4 MPa) and durations (5, 30, 60 min) (**A**–**C**). Data is shown as means ± SD (*n* = 3); two-way ANOVA with Tukey’s post hoc test was performed. Statistical comparisons were performed relative to LPS-treated controls; *p < 0.05, **p < 0.01, ***p < 0.001, ****p < 0.0001.

To evaluate any changes in cytotoxicity induced by ultrasound in LPS-treated microglia, we measured the LDH release. LDH is a stable cytoplasmic enzyme that is present at high levels when cells undergo damage or stress. As expected, LPS significantly increased LDH release compared to untreated controls (*p* < 0.0001; Fig. 3A–C), while ultrasound was able to reduce these elevated cytotoxic levels across most parameter sets. At 0.5 MHz, LDH levels decreased at 0.1 MPa and 0.2 MPa after 5 and 30 min of treatment, and at 0.4 MPa across all treatment durations (Fig. 3A). Similarly, at 1 MHz, reductions in cytotoxicity were observed at 0.1 MPa after 5 and 30 min of ultrasound, at 0.2 MPa for all durations, and at 0.4 MPa after 60 min of ultrasound (Fig. 3B). Notably, 2 MHz parameter sets produced the most consistent reductions in LDH release across all the parameters tested (Fig. 3C).

Overall, the ultrasound parameters that reduced inflammatory and cytotoxic responses most effectively in microglia were 0.5 MHz at 0.2 MPa for 5 min, and 2 MHz at 0.1 and 0.2 MPa for 5 min (Figs. 1 and 3). The 0.5 MHz parameter set was selected for further investigation due to its greater clinical relevance for neurological applications, as lower frequencies penetrate the skull more effectively and can reach deeper brain regions.

### Ultrasound modulates NF-κB-associated cytokines and enhances anti-inflammatory gene expression in microglia for up to 72 h

**Fig. 4:**
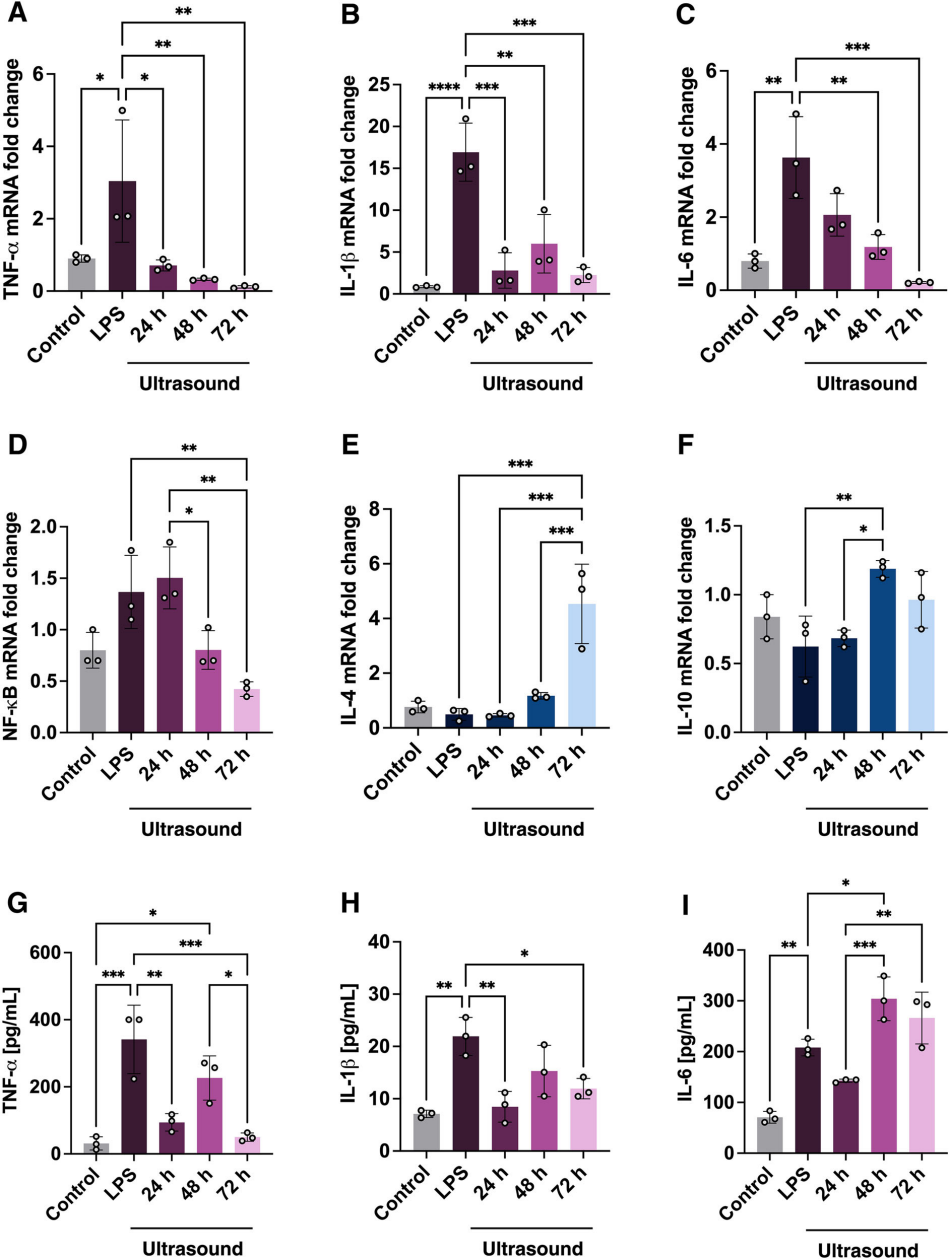
Ultrasound modulates NF-κB-associated cytokines and enhances anti-inflammatory gene expression in microglia for up to 72 h. Relative mRNA expression of pro- and anti-inflammatory genes (**A**–**F**) and protein levels of TNF-α, IL-1β, and IL-6 (**G**–**I**) were measured in LPS-treated microglia at 24, 48 and 72 h following ultrasound at 0.5 MHz and 0.2 MPa for 5 min. Data is shown as means ± SD (*n* = 3); one-way ANOVA with Tukey’s post hoc test. Statistical comparisons were performed relative to control and LPS treated groups; *p < 0.05, **p < 0.01, ***p < 0.001.

To explore the time-dependent changes of ultrasound on microglia, we analysed the mRNA expression of pro- and anti-inflammatory genes 24, 48 and 72 h after ultrasound treatment using the selected parameter set (0.5 MHz at 0.2 MPa for 5 min; Fig. 4). Ultrasound significantly reduced TNF-α gene expression compared with LPS-treated microglia 24 h after treatment (*p* < 0.05), with the effect persisting at 48 and 72 h (*p* < 0.01; Fig. 4A). Similarly, IL-1β and IL-6 mRNA levels were significantly downregulated for up to 72 h, suggesting a sustained anti-inflammatory response (*p* < 0.001; Fig. 4B, C).

To investigate the underlying mechanisms, we assessed NF-κB gene expression, a key transcriptional regulator of TNF-α, IL-1β and IL-6. NF-κB expression was significantly decreased 72 h after ultrasound, consistent with the reductions observed in pro-inflammatory cytokines (Fig. 4D). These findings suggest that NF-κB signalling contributes to the ultrasound-induced modulation of these pro-inflammatory cytokines.

Additionally, anti-inflammatory IL-10 and IL-4 mRNA levels were upregulated following ultrasound treatment. IL-4 expression was significantly increased 72 h after (*p* < 0.001; Fig. 4E) and IL-10 expression was significantly increased 48 h after ultrasound compared with LPS-treated microglia (*p* < 0.01; Fig. 4F). These results indicate that ultrasound can induce a transcriptional shift by downregulating NF-κB-mediated pro-inflammatory genes while promoting anti-inflammatory changes associated with immune resolution.

To determine whether these gene expression changes translated to the protein level, we measured the concentration of TNF-α, IL-1β and IL-6 cytokines 24, 48 and 72 h after ultrasound treatment. TNF-α and IL-1β protein levels were significantly lower 24 and 72 h after ultrasound compared with LPS-treated microglia (Fig. 4G, H), whereas IL-6 protein levels did not change at the 24 and 72 h time points but were found to increase 48 h after treatment (Fig. 4I).

### Ultrasound attenuates TNF-α in the hippocampus of an LPS mouse model of neuroinflammation

**Fig. 5:**
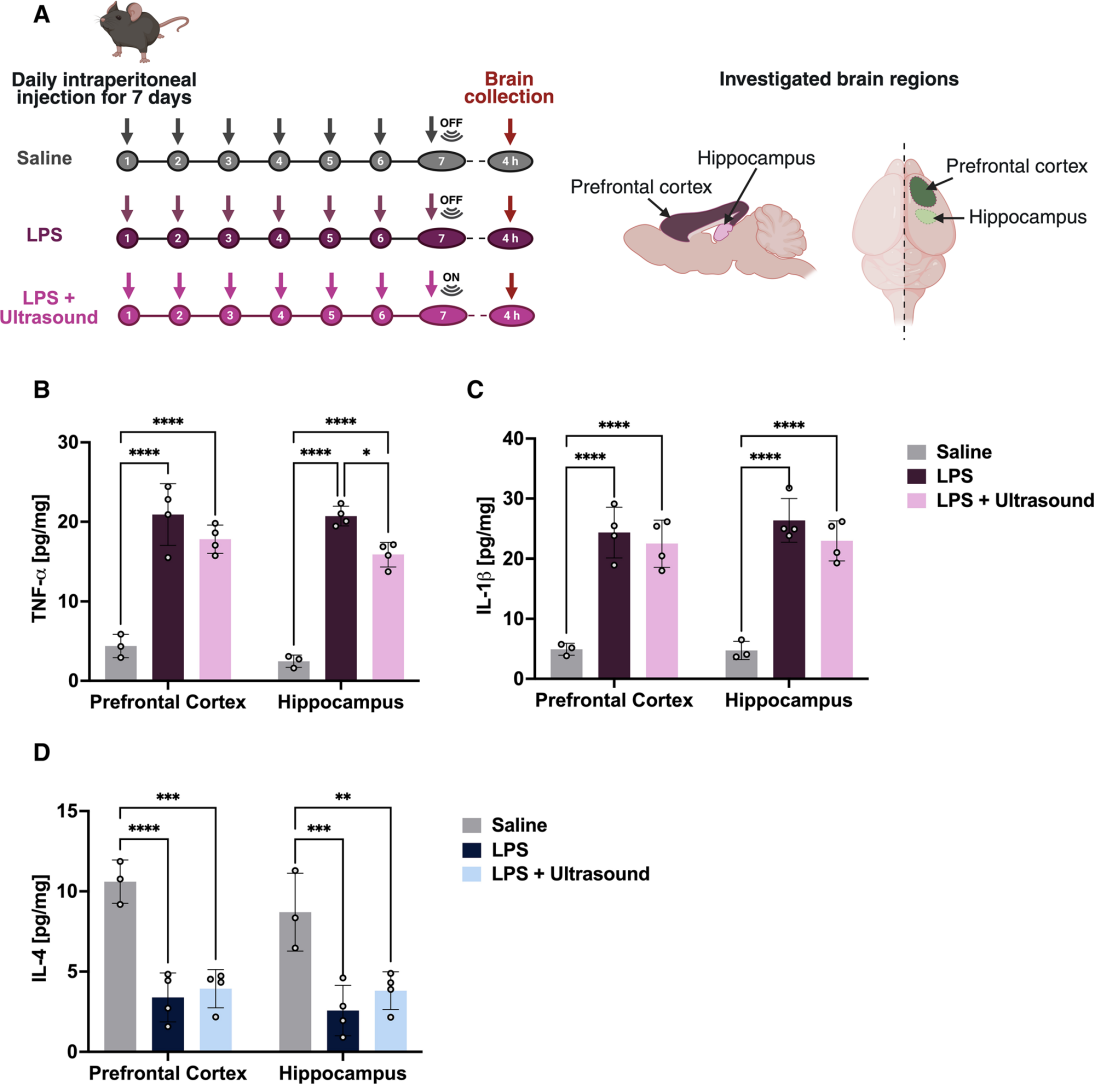
Ultrasound attenuates TNF-α in the hippocampus of an LPS mouse model of neuroinflammation. Schematic of the experimental procedure and brain regions analysed are shown (**A**). Protein levels of TNF-α, IL-1β, and IL-4 in the prefrontal cortex and hippocampus are shown for saline, LPS and LPS + ultrasound-treated mice 4 h post-treatment (**B–D**). Data is shown as means ± SD (saline *n* = 3, LPS and LPS + ultrasound *n* = 4); two-way repeated-measures ANOVA with Tukey’s post hoc test was performed. Statistical comparisons were made relative to saline and LPS controls; *p < 0.05, **p < 0.01, ***p < 0.001, ****p < 0.0001.

To determine whether the in vitro effects of ultrasound translate in vivo, we evaluated the selected parameter set (0.5 MHz at 0.2 MPa) in a mouse model of sustained inflammation. Mice received daily intraperitoneal injections of LPS or saline for 7 consecutive days to induce neuroinflammation and activate microglia (Fig. 5A)^27^. On day 7, mice in the LPS + Ultrasound group received a single whole-brain ultrasound treatment, whereas the saline and LPS-only groups underwent sham treatments. Four hours post-treatment, brains were collected for morphological and protein analysis (Fig. 5A). All the in vivo data presented were obtained from female mice.

TNF-α, IL-1β and IL-4 protein levels were quantified in the prefrontal cortex and hippocampus (Fig. 5A). LPS administration significantly increased TNF-α and IL-1β levels, indicating an enhanced inflammatory response (*p* < 0.0001; Fig. 5B−C). Ultrasound treatment reduced TNF-α levels in the hippocampus significantly compared with LPS-only mice (*p* < 0.05; Fig. 5B), although levels remained higher than in saline controls. IL-1β and IL-4 levels, on the other hand, were not significantly altered by ultrasound at this early 4 h time point compared to LPS-treated mice (Fig. 5B−D).

### Ultrasound induces changes in microglial morphology

**Fig. 6:**
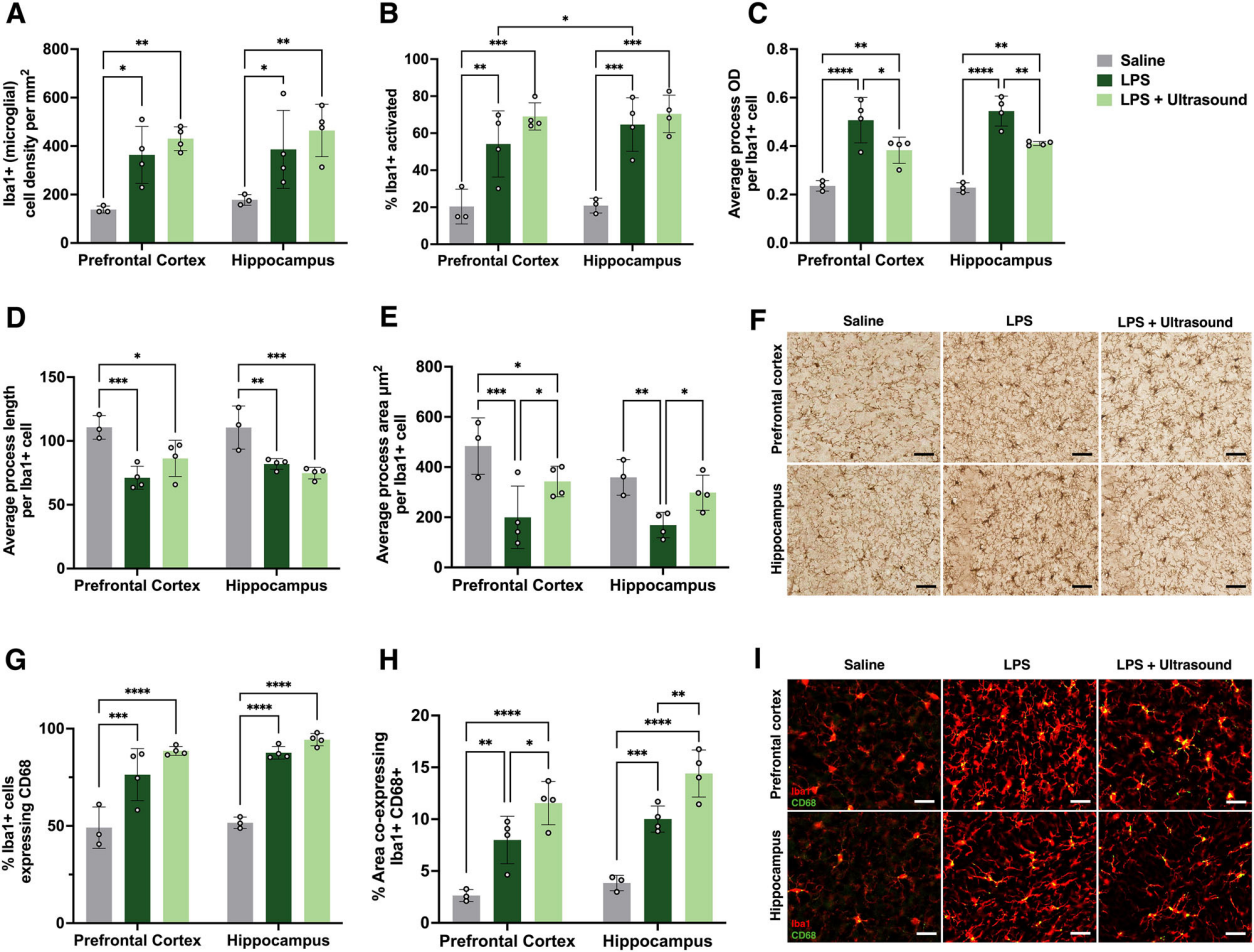
Ultrasound induces changes in microglial morphology. Quantification of Iba1+ microglia shown for the prefrontal cortex and hippocampus using DAB staining: **A** Iba1+ cell density (cells/mm^2^), **B** percentage of activated Iba1+ microglia, **C** average process optical density (OD) per Iba1+ cell, **D** average process length per Iba1+ cell, and **E** average process area (µm^2^) per Iba1+ cell. **F** Representative DAB-stained images of microglia in saline, LPS and LPS + ultrasound-treated mice at 4 h post-treatment (scale bar at 100 µm). Quantification of Iba1 + CD68 immunofluorescence quantification shown: **G** percentage of Iba1+ cells expressing CD68, **H** percentage of area co-expressing Iba1 + CD68+ and **I** representative immunofluorescence images of microglia in saline, LPS and LPS + ultrasound-treated mice 4 h post-treatment (scale bar at 100 µm). Data shown is as means ± SD (saline *n* = 3, LPS and LPS + ultrasound *n* = 4). Two-way repeated measures ANOVA with Tukey’s post hoc test was performed with statistical comparisons done relative to saline and LPS groups; *p < 0.05, **p < 0.01, ***p < 0.001, ****p < 0.0001.

We next assessed microglial morphology by immunostaining brain sections for Iba1, a morphological marker of microglia, in the prefrontal cortex and hippocampus (Fig. 6A–F). LPS treatment alone markedly altered microglial morphology. Compared with saline controls, LPS increased the density of Iba1-positive microglia in both regions (*p* < 0.05; Fig. 6A) and significantly elevated the proportion of cells classified as “activated” by the HALO software (*p* < 0.01; Fig. 6B). LPS also increased the average optical density (OD) of Iba1 staining per microglial cell, indicating enhanced Iba1 expression (*p* < 0.0001; Fig. 6C), and induced shortening and retraction of processes, reflected by decreased average process length and area (*p* < 0.001 and *p* < 0.01, respectively; Fig. 6D, E). Morphologically, LPS-treated microglia exhibited enlarged cell bodies with thick, retracted processes and multiple spiny protrusions, giving them a “bushy” appearance characteristic of a reactive phenotype (Fig. 6F).

Ultrasound treatment partially mitigated several of these LPS-induced changes. In both regions, ultrasound reduced the average Iba1 OD compared with LPS-only mice (*p* < 0.05 and *p* < 0.01; Fig. 6C), although, at this time point (4 h after ultrasound), values did not return to baseline saline levels. Ultrasound also increased the average process area per microglial cell relative to LPS treatment alone (*p* < 0.05; Fig. 6E), suggesting partial recovery of microglial branching complexity.

We next assessed microglial lysosomal activation by co-staining for Iba1 and CD68, a lysosomal protein marker (Fig. 6G–I). LPS led to a significant increase in both the proportion of microglia expressing CD68 and the CD68-positive area in both the prefrontal cortex and hippocampus compared with saline controls (Fig. 6G, H), consistent with an inflammatory response. Ultrasound did not alter the proportion of CD68-positive microglia relative to LPS alone (Fig. 6G), indicating no shift in the number of cells engaging lysosomal pathways at this time points. However, ultrasound significantly increased the area of microglia expressing CD68 in both brain regions compared with LPS (Fig. 6H), pointing to an increase in lysosomal protein content per microglial cell, which could potentially be associated with phagocytic activity.

Together, these findings indicate that LPS induces pronounced morphological changes of microglia, and that ultrasound treatment shows promise in partially reversing these structural changes, despite not fully attenuating lysosomal activation to saline levels at this early time point (Fig. 6G, H).

## Discussion

Neuroinflammation contributes to the progression of numerous neurological diseases, with microglia playing a key regulatory role. Microglia promptly respond to perturbations in their microenvironment by releasing a range of cytokines that can either restore homeostasis or exacerbate disease^9^. In this study, we aimed to determine whether ultrasound could modulate microglial-mediated inflammation in both in vitro and in vivo models. By screening a range of ultrasound parameters, we found that distinct parameters differentially regulate the expression of pro-inflammatory cytokines in LPS-treated microglia. Our in vitro results revealed that the modulatory effects of ultrasound on microglia depend on the centre frequency applied. Among the frequencies tested, 2 MHz consistently produced the most pronounced reduction in inflammation, even at the lowest pressure (0.1 MPa) and shortest treatment duration (5 min). Reductions were also observed at 0.5 and 1 MHz, although only under specific parameter combinations. Importantly, the reduction in inflammation induced by ultrasound was not associated with increased cytotoxicity, as confirmed by the LDH assay.

Despite the efficacy of 2 MHz treatments in reducing inflammation, we selected the 0.5 MHz parameter set for further investigation due to its greater tissue penetration, which is more clinically relevant for transcranial applications. Lower frequencies are better suited at overcoming the attenuation of the skull, as longer wavelengths lose less energy when propagating through bone^28^, allowing for deeper penetration into the brain. Higher frequencies may be preferable for peripheral tissue applications, where skull attenuation is not a limiting factor. For subsequent gene and protein analysis, we selected 0.5 MHz at 0.2 MPa for 5 min as a clinically relevant parameter set for transcranial brain applications.

Following ultrasound treatment with the selected parameter set, we observed a significant downregulation of pro-inflammatory cytokine genes and an upregulation of anti-inflammatory genes. These findings indicate that ultrasound not only reduces inflammation but also promotes a shift towards tissue repair and regeneration. The observed downregulation of NF-κB gene expression also suggests that ultrasound modulates this pathway at the transcriptional level.

Mice exposed to seven consecutive days of LPS exhibited significant changes in body weight, pro-inflammatory cytokine levels, and microglial morphology (Figs. 5 and 6). Using the selected ultrasound parameters, we found a significant reduction in hippocampal TNF-α levels and morphological changes in microglia as early as 4 h after a single ultrasound treatment. Following systemic LPS challenges, microglia can respond rapidly and typically display hypertrophic changes, characterised by shortened, thickened processes and increased Iba1+ process OD^7,8,29^. In the current study, we provided evidence that ultrasound treatment can reduce Iba1+ process OD and increase the average process area compared with the LPS-treated group. These early morphological changes suggest that ultrasound may restore microglial structure towards a more ramified and homeostatic state that is supportive of inflammation resolution. Given that our in vivo analyses were conducted in an acute timeframe, further changes at later stages should be investigated in future studies.

Our results align with previous studies showing that ultrasound can modulate inflammation and enhance protective functions in microglia. In vitro studies have shown that ultrasound attenuates TNF-α, IL-1β and IL-6 protein levels in LPS-treated BV2 microglia^19^ and increases arginase-1 and IL-10 gene expression^24^. Building on these findings, our work demonstrates that ultrasound regulates microglial cytokine expression and reduces cytotoxicity through parameter-dependent mechanisms. Additionally, NF-κB has been identified as a potential therapeutic target due to its central role in mediating inflammation^30,31^. Our results align with previous studies, which also demonstrate a reduction in NF-κB expression following ultrasound^17,19^. This suggests that the modulation of this pathway may contribute to the anti-inflammatory effects of the treatment. The underlying mechanisms likely involve the activation of mechanosensitive ion channels, which respond to ultrasound mechanical forces and influence downstream signalling pathways. Ultrasound-induced mechanical forces can activate channels like PIEZO1/2 and TRPV4, leading to calcium influx and modulation of immune signalling pathways^32,33^. Microglia express several mechanosensitive ion channels, including PIEZO1, that coordinate both physiological and pathological processes^34,35^. It is therefore plausible that ultrasound modulates microglial immune responses through the activation of mechanosensitive ion channels, converging on transcriptional regulators such as NF-κB. The parameter-dependent effects observed in vitro, particularly at the 2 MHz centre frequency, suggest that differences in acoustic radiation force under these conditions may contribute to the observed cellular responses^36^. At higher centre frequencies, increased acoustic absorption in soft tissue can lead to greater radiation force-induced displacement for a given acoustic intensity, which can increase mechanotransductive effects at the cellular level^36–38^. This may explain the stronger bioeffects observed when microglial cells were treated at 2 MHz as compared with 0.5 MHz. Additionally, acoustic pressure and stimulation duration may also modulate the cellular membrane and ion channel activity. In addition to the activation of mechanosensitive ion channels, our findings may also involve heat shock proteins that help protect cells from damage. Previous studies have reported that low-intensity pulsed ultrasound can activate these proteins, such as HSP70 and HSP90, which promote cellular repair mechanisms by reducing the production of pro-inflammatory cytokines like IL-1β and TNF-α^39,40^.

Studies have shown that repeated ultrasound treatments can promote the clearance of neurotoxic debris after stroke injury^41^ and reduce hippocampal TNF-α and IL-1β protein levels in LPS-treated mice^42^. Our data reveals a significant reduction in hippocampal TNF-α levels following a single ultrasound treatment. At this time point, the protein levels of IL-1β and IL-4 remained unchanged; however, assessing these levels after repeated sessions or at later time points could reveal differences in these cytokines. Additionally, ultrasound was applied for only 6 seconds (s) per targeted brain region, and longer sonication durations could potentially further enhance the modulation effects.

Regional brain effects and microglial heterogeneity should also be considered when interpreting our results. The hippocampus, in particular, is among the brain regions most susceptible to LPS-induced inflammation^43,44^, with increased expression of pro-inflammatory cytokines and pronounced microglial responses compared to the cortex^45^. Consistently, in our LPS model, we observed a higher proportion of morphologically activated Iba1+ microglia, characterised by an enlarged soma and retracted processes in the hippocampus compared with the prefrontal cortex (Fig. 6B). Microglia display region-specific morphologies as well as distinct gene and protein expression profiles across different brain regions^46,47^. Compared with cortical microglia, those in the hippocampus tend to exhibit a hyper-ramified morphology and greater lysosomal and phagocytic activity^48^. These characteristics may render the hippocampus more responsive to therapeutic interventions such as ultrasound. The hippocampus is more vulnerable in several neurological diseases, including Alzheimer’s disease, where efficient clearance through phagocytosis of pathological proteins such as amyloid-β and neurofibrillary tangles is critical to slowing disease progression^49^. In our histology data, we observed an increase in the area of microglia positive for CD68 after ultrasound treatment in both the hippocampus and prefrontal cortex. This enhanced lysosomal activity in the LPS brain could potentially indicate that these cells are primed for phagocytosis of cellular debris. To determine whether ultrasound promotes microglial phagocytosis, future studies should include neuronal and synaptic markers alongside CD68 measurements to identify engulfed components within microglia.

For our in vitro experiments, we used an immortalized BV2 microglial cell line to assess the direct effects of ultrasound parameters. However, immortalized cultures do not fully reproduce the complexity of primary or human microglia, which display greater heterogeneity and context-dependent phenotypes. To enhance therapeutic relevance, future studies should incorporate induced pluripotent stem cell-derived human microglial models. Given the functional diversity of microglia, future investigations should also employ multi-omic approaches such as transcriptomics and proteomics to obtain a more comprehensive understanding of microglial responses to ultrasound. Moreover, microglia in vivo engage in cross-communication with other brain cells, including astrocytes and neurons, which play key roles in modulating the immune responses in homeostasis and in disease^50,51^. The absence of this interaction of cells in our in vitro model lies as a limitation, and therefore, to enhance the biological relevance, future studies should incorporate co-culture systems that can better recapitulate the brain’s cellular environment. Additionally, LPS stimulation is a well-established model for studying sustained inflammation, however, it does not fully recapitulate the inflammation driven by genetic or pathological markers, such as amyloid or tau, which is found in neurodegenerative diseases^52^. In particular, LPS lacks the progressive accumulation of amyloid and tau pathology and progressive neuronal and synaptic degeneration^52^. Future studies will incorporate disease-relevant models, including transgenic Alzheimer’s mice or amyloid-β stimulation in vitro, which would be essential to extend these findings. In the in vivo study, we applied ultrasound for just 6 s per target to scan the entire brain. This method aligns with previous studies that have reported the modulation of microglia with ultrasound with this short exposure time^53,54^. While this approach minimised anaesthesia duration and enabled whole-brain targeting, future studies will explore whether longer sonication times or repeated treatments could enhance the effects observed on cytokines and microglial morphology. Additionally, a 4 h post-treatment time point was chosen to investigate whether ultrasound could modulate early immune responses. Previous studies show that LPS-induced neuroinflammation can lead to rapid morphological and cytokine changes in the hippocampus within hours of administration^55^. Future work will also assess additional time points, such as 24 and 72 h as per our in vitro experiments, which could reveal further changes in cytokine activity that were not captured during this acute phase. A further limitation of the in vivo study was the use of only female mice. Previous evidence shows that female microglia display a lower baseline inflammatory response and enhanced tissue repair mechanisms compared to males, which may influence the degree of immune modulation achievable with ultrasound^56,57^. Future studies should incorporate animals of both sexes, particularly when mapping parameter-mediated effects of ultrasound. This is also important for clinical translation, especially in the context of diseases where sex differences in disease prevalence and therapeutic outcome are known. Larger sample sizes will also strengthen the robustness of future findings.

Overall, our study adds to the growing evidence that ultrasound can modulate neuroinflammation, supporting its potential as a non-invasive therapeutic strategy for neuroinflammatory diseases. Our results suggest that ultrasound can help restore immune homeostasis without causing widespread immunosuppression, which remains an ongoing clinical challenge.

## Methods

### Cell culture

**Fig. 7:**
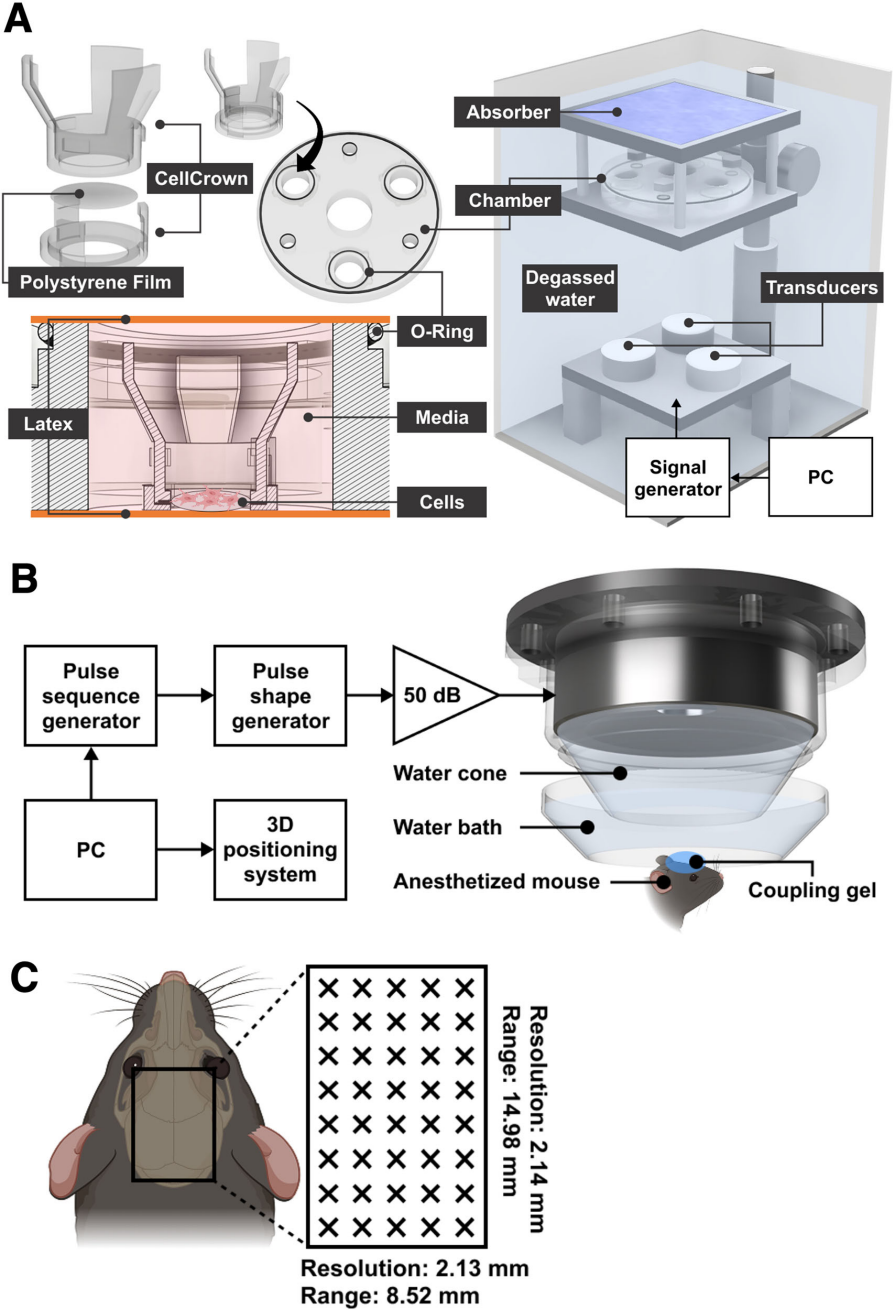
In vitro and in vivo experimental setups. **A** In vitro experiments were performed by culturing cells in CellCrown™ inserts mounted on a polystyrene film within a latex-sealed device filled with media. The device was placed above three unfocused transducers and submerged in degassed water to ensure acoustic coupling. The transducers were driven by a 4-channel signal generator controlled by a PC software to modulate acoustic pressure, pulse repetition frequency, duty cycle, and treatment duration. **B** In vivo experiments were performed with a 0.5 MHz focused transducer mounted on a 3D positioning system. The transducer was coupled to the head via a water-filled cone and parafilm-bottomed bath containing deionised water, with ultrasound gel applied for acoustic coupling. Ultrasound pulses were emitted from the transducer driven by a function generator through a 50-dB power amplifier. **C** The whole mouse brain was treated with ultrasound by moving the transducer within a grid of 8.52 × 14.98 mm in size. Figures were created with Fusion 360, BioRender and Figma.

BV2 cells (ATCC^®^), a murine microglial cell line, were obtained from Professor Elisabetta Ferraro from the University of Pisa, Italy. Cells were maintained in Dulbecco’s modified Eagle’s medium (DMEM, Sigma-Aldrich) supplemented with 10% fetal bovine serum (FBS, Sigma-Aldrich), 100 μL/mL penicillin, and 100 μg/mL streptomycin (Sigma-Aldrich). Upon 90% confluency, the cells were trypsinized, centrifuged, counted and seeded at a cell density of 1 × 10^4^ cells/well on a thin polystyrene film (Goodfellow) mounted within a CellCrown (Scaffdex) in a 24 plate (Fig. 7A). The cells were divided into three groups: (i) control, (ii) LPS and (iii) LPS + ultrasound. BV2 cells in groups (ii) and (iii) were treated with 1 µg/mL LPS for 24 h to establish a cellular model of neuroinflammation. Next, the cells in group (iii) (LPS + ultrasound) were treated with various ultrasound parameters. In particular, the group consisted of 9 sub-groups based on the required combinations of centre frequency (0.5, 1, 2 MHz), acoustic peak-to-peak pressure (0.1, 0.2, 0.4 MPa) and treatment duration (5, 30, 60 min). After 24 h, the supernatant was collected for analysis.

### In vitro ultrasound treatment

A custom in vitro ultrasound system was used to perform the ultrasound treatment of BV2 cells^58^. Three piezoelectric unfocused transducers with centre frequencies of 0.5 MHz (44 mm diameter), 1 MHz (23 mm diameter) and 2 MHz (23 mm diameter) were used for the study (Precision Acoustics, UK). The transducers were powered by a 4-channel signal generator (2 W/channel) controlled by software for setting the acoustic pressure, duty cycle, pulse repetition frequency and treatment duration (Image Guided Therapy, France; Fig. 7A). Duty cycle and pulse repetition frequency were kept constant at 20% and 1 kHz, respectively. Temperature measurements were determined using a fine-wire type T thermocouple probe positioned inside the wells (T.M. Electronics, UK, resolution of 0.1 °C). The spatial-peak pulse-average intensity (Isppa) was calculated using the equation ${I}_{{\rm{sppa}}}=\frac{{p}_{{\rm{rms}}}^{2}}{\rho c}$, and the spatial-peak temporal average intensity (Ispta) was calculated by multiplying ${I}_{\mathrm{sppa}}$ by the duty cycle (20%). A summary of calculated intensities for each acoustic pressure used in this study is provided in Table S1.

### Animals

Eleven female C57BL/6 mice (8–9 weeks old, 19.3 ± 1.2 g, Charles River, Cambridge, UK) were used in this study. All mice were housed in a 12 h light/12 h dark cycle with free access to food and water, and were acclimatised for seven days before any procedure. This study was designed as a pilot study and all animals were randomly divided into three groups: (i) saline *n* = 3, (ii) LPS *n* = 4 and (iii) LPS + ultrasound *n* = 4. Mice in groups (ii) and (iii) were intraperitoneally injected with LPS (Sigma-Aldrich, Cat. O111:B4) at a dose of 0.75 mg/kg for seven consecutive days, while mice in group (i) were administered with an equal volume of 0.9% saline NaCl (Sigma-Aldrich). LPS and saline injections were administered between 09:00 and 11:00 during the animals’ active (dark) phase of a reversed light/dark cycle. Ultrasound or sham treatment was applied on the seventh day, 4 h after the final saline or LPS injection. All mice were weighed daily prior to any procedures and were housed under identical conditions throughout the study. No animals or data points were excluded from the analysis. All animal experiments were conducted in accordance with the Animals Scientific Procedures Act 1986 and approved by UK Home Office and Imperial College London’s Welfare and Ethical Review Body (PBDB8AA12).

### In vivo ultrasound treatment

For the in vivo experiments, mice were anaesthetised with 2% vaporised isoflurane (Zoetis UK Limited, London, UK) with oxygen (Harvard Apparatus, Cambridge, UK). The fur on the head was shaved using an electric trimmer and then depilatory cream. Next, the mouse’s head was positioned within a stereotaxic frame (45° ear bars; World Precision Instruments, Hertfordshire, UK). A single-element spherical focused ultrasound transducer (centre frequency 0.5 MHz; focal depth: 39.49 mm, active diameter: 22.60 mm, part number: H-107; Sonic Concepts, Bothwell, WA, USA) was used to emit the ultrasound (Fig. 7B). The parameters used were 0.5 MHz centre frequency, 0.2 MPa acoustic pressure, 1 kHz pulse repetition frequency, 200 μs pulse length, and 6 s sonication duration for each targeted point within a grid (8.52 mm × 14.98 mm) to cover the entire brain of the mouse (Fig. 7C). A 6 s sonication duration per targeted point was chosen based on existing ultrasound studies^53,54^, and due to longer durations not being as feasible given that the whole brain was targeted. The transducer was mounted onto a 3D positioning system (Velmex, Bloomfield, NY, USA) to move the ultrasound focus in the *x* and *y* directions to cover the entire brain. A cone mounted onto the transducer was filled with deionised water and placed into a parafilm-bottomed bath also filled with deionised water (Fig. 7B). This was placed on the head of the mouse with ultrasound gel for coupling to ensure propagation of the ultrasound into the brain. Ultrasound pulses were generated by a function generator (33500B series; Agilent Technologies, Santa Clara, CA, USA), with the signal driven by a 50-dB power amplifier (2100 L Electronics and Innovation, Rochester, NY, USA) (Fig. 7B). Control mice treated with saline and LPS received the same duration of anaesthesia as those treated with LPS + ultrasound and were placed under the transducer, without turning on the amplifier. 4 h after treatment, all mice were injected intraperitoneally with pentobarbital before being transcardially perfused with 30 mL of ice-cold phosphate-buffered saline (PBS; Gibco). The brains were extracted from the skull and split into two hemispheres. The left hemisphere was fixed with 4% formalin (Sigma-Aldrich) for 24 h and cryoprotected in 30% sucrose in PBS at 4 °C. The right hemisphere was further dissected into prefrontal cortex and hippocampus regions, which were snap frozen in dry ice and stored at −80 °C for protein analysis.

### Acoustic simulations

To estimate potential temperature increases in our in vivo experiments, we simulated the propagation pathway of our ultrasound pulses using a k-Wave MATLAB toolbox^59^. The anatomical skull geometry of a mouse was derived from a publicly available micro-CT murine skull, voxelised and rescaled to match the simulation grid resolution of 0.231 mm, corresponding to 13 points per wavelength at 0.5 MHz^60^. The Courant–Friedrichs–Lewy number was set to 0.1 and the parameters of the simulations were set to 0.5 MHz centre frequency, 0.2 MPa pressure at the acoustic focus in water, 200 μs pulse length, 1 kHz pulse repetition frequency, and 6 s sonication duration. The transducer was positioned perpendicularly to the skull surface, so that its natural acoustic focus coincided with the centre of the mouse brain. The mouse skull was treated as a homogeneous material, while the brain and soft tissues were assumed to have the acoustic properties of water. In addition, perfect acoustic coupling was assumed between the transducer and the skull, representing a conservative worst-case scenario with maximum transmission efficiency (Table S2).

Temperature was evaluated at five spatial positions within the skull, corresponding to the centre and the four vertices of the grid used in the in vivo experiments. To achieve this, the transducer was repositioned at each location so that its geometric focus aligned with the respective target point inside the brain. A 200 µs pulse was used to simulate the acoustic field. The peak pressure was converted to root-mean-square pressure to compute pulse average acoustic intensity: ${I}=\frac{{p}_{{rms}}^{2}}{\rho \cdot c}$. In the formula, $\rho$ is the medium’s density and $c$ is the speed of sound in the medium. Heat deposition was then defined as:$\,Q=2\cdot {\alpha }_{{np}}\cdot I\cdot D.{D}$ is the duty cycle, and ${\alpha }_{{np}}$ is the absorption coefficient in Nepers per metre, obtained from the frequency-dependent attenuation input. This heat source was passed to the k-Wave thermal solver and used to simulate heating over a 6 s sonication period with a duty cycle of 20%, corresponding to 200 µs pulses at 1 kHz pulse repetition frequency.

These simulations showed negligible thermal effects, with peak temperature rises remaining below 0.02 °C (Fig. S1). Specifically, the mean temperature increase in the skull was 0.0134 ± 0.0048 °C, while in the surrounding regions (brain and scalp), the increase was 0.0117 ± 0.0038 °C, suggesting that thermal effects were minimal for the selected treatment parameters. The thermal dose was estimated using k-Wave to calculate cumulative equivalent minutes at 43 °C (CEM43) at the focus of the ultrasound beam. Based on these simulations, the in vivo sonication is likely to produce a negligible thermal dose (0.000024 min) average at focus points within the mouse brain, supporting the conclusion that thermal effects were not significant under the tested neuromodulation conditions^26^.

### Enzyme-linked immunosorbent assay (ELISA)

BV2 cell supernatant was collected 24 h after ultrasound treatment, and cytokine levels were analysed using mouse TNF-α (Cat. KE10002), IL-1β (Cat. KE10003), and IL-6 (Cat. KE10091) ELISA kits (Proteintech, UK), according to the manufacturer’s instructions. The absorbance was measured using a multilabel plate reader (Victor, PerkinElmer, Waltham, MA, USA), recorded at 450 nm. The prefrontal cortex and hippocampus were homogenised in RIPA lysis buffer (Thermo-Fisher, Cat. 89901) with proteinase tablets (Thermo-Fisher, Cat. A32955). Samples were centrifuged at 15,000 × *g* for 15 min. The supernatant was collected, and the protein concentration was determined using a Pierce BCA protein reagent kit (Thermo-Fisher, Cat. 23225). ELISAs (Proteintech, UK) for TNF-α (Cat. KE10002), IL-1β (Cat. KE10003) and IL-4 (Cat. KE10010) were performed according to the manufacturer’s instructions. The absorbance was measured using a microplate reader (Varioscan, Thermo-Fisher, UK), recorded at 450 nm.

### Lactate dehydrogenase (LDH) assay

LDH levels in the experimental samples were detected using a CyQUANT^TM^ LDH Cytotoxicity Assay kit (Thermo-Fisher, Cat. C20301), according to the manufacturer’s instructions. The assay kit quantified LDH levels by LDH-induced catalysis of the enzymatic conversion of lactate to pyruvate via NAD+ reduction to NADH. The absorbance readings taken using a multilabel plate reader (Victor, PerkinElmer, Waltham, MA, USA) at 680 nm were subtracted from those at 490 nm.

### Real-time quantitative reverse transcription polymerase chain reaction (RT-qPCR)

Total RNA was isolated from the BV2 cells using the ReliaPrep™ RNA Cell Miniprep System according to the manufacturer’s instructions at 24, 48 and 72 h post-ultrasound (Promega, Cat. Z6011). Next, a NanoDrop spectrophotometer was used to assess the concentration and the purity of the RNA. Total RNA was then reverse transcribed to cDNA using the PrimeScript™ RT Master Mix (Takara, Cat. RR036A), according to the manufacturer’s protocol. Real-time qRT-PCR was performed using PowerUp™ SYBR™ Green Master Mix (Thermo-Fisher, Cat. A25742) in a Rotor-Gene Q (Qiagen, Hilden, Germany) following the manufacturer’s instructions, for the following genes: TNF-α, IL-1β, IL-6, NF-κB, IL-10 and IL-4. The mRNA levels of these genes were calculated relative to the glyceraldehyde-3-phosphate dehydrogenase (GAPDH) house-keeping gene using the 2^−ΔΔCT^ method and expressed as fold change, equivalent to normalization with respect to the control group^61^. The primers used are listed in Table S3.

### Histology

Free-floating sagittal brain sections (30 μm) were obtained using a cryostat (CryoStar NX70; Thermo-Fisher). The blade temperature was set to −14 °C and −12 °C, respectively, and the sections were collected in a 24-well plate with 0.05% sodium azide (Sigma-Aldrich) in PBS. For immunostaining with 3,3-diaminobenzidine (DAB), sections were washed in tris-buffered saline (TBS; Sigma-Aldrich), then permeabilised with 1% hydrogen peroxide (Sigma-Aldrich) in 0.25% TBS Triton-X (Sigma-Aldrich). Sections were blocked using 10% FBS and 1% bovine serum albumin (BSA; Sigma-Aldrich) in 0.1% TBS Triton-X for 2 h. Then, the brain sections were incubated using 1:1000 rabbit anti-Iba1 (019-1941; Fujifilm WAKO Chemicals, USA) primary antibody in 2% FBS, 0.2% BSA and 0.02% TBS Triton-X overnight at room temperature. The sections were washed with TBS and incubated with 1:200 goat anti-rabbit IgG (BA-1000-1.5; Vector Laboratories) secondary biotinylated antibody in 2% FBS, 0.2% BSA and 0.02% TBS Triton-X for 2 h at room temperature. Sections were washed 3 times with TBS and then PBS, followed by signal amplification with avidin biotin horseradish peroxidase complex (PK-6100; Vector Laboratories). The stain was visualised with DAB (SK-4105; Vector Laboratories), and the reaction was stopped after 20 s using distilled water. Brain sections were mounted onto microscope slides (VWR European, Cat. 631-0108) in a PBS bath and left overnight to dry. Finally, the slides were dehydrated in distilled water, 70%, 90%, 100% ethanol (Sigma-Aldrich) and xylene (Sigma-Aldrich) and then cover-slipped with DPX mounting medium (Sigma-Aldrich). Images were acquired with a digital slide scanner (Aperio AT2; Leica) using a 20X objective. All images were analysed using HALO quantitative image analysis software (Indica Labs). HALO parameters used for the microglial analysis are listed in Table S4.

For immunofluorescence, brain sections were washed with TBS and then permeabilised with 0.25% TBS Triton-X. Sections were blocked using 10% FBS and 1% BSA in 0.1% TBS Triton-X for 2 h. Next, brain sections were incubated with 1:1000 rabbit anti-Iba1 and 1:500 rat anti-CD68 (MCA1957-MO; Bio-Rad, UK) primary antibodies overnight at 4 °C. After, the sections were washed 5 times in TBS, followed by incubation with 1:1000 donkey anti-rabbit (ab150075; Alexa Fluor 647; Abcam, UK) and 1:1000 goat anti-rat (ab150157; Alexa Fluor 488; Abcam, UK) secondary antibodies with 2% FBS, 0.2% BSA in 0.02% TBS Triton-X for 2 h. Following incubation, the sections were washed 5 times with TBS and mounted onto microscope slides with mounting media (Abcam, Cat. AB104135). Images were acquired using a MICA microhub fluorescent microscope (Leica Microsystems, UK) at 20X magnification using filters with excitation at 653 nm and emission at 669 nm to image Iba1 and excitation 499 nm and emission at 519 nm for CD68 imaging. All images were processed using an imaging software on MATLAB.

### Statistical analysis

Data was normalised according to the Shapiro-Wilk test, and significant differences were evaluated using one-way or two-way ANOVA followed by Tukey’s post hoc multiple comparison tests. All statistical analysis was performed using GraphPad Prism (Prism 10, La Jolla, California, USA), and the criteria of significance was set at *p < 0.05, **p < 0.01, ***p < 0.001, ****p < 0.0001. Values shown are the mean and standard deviation of the mean (mean ± SD).

## Supplementary information

Supplemental Information

Data S1

## Data Availability

The data supporting the findings are provided in Data S1. Additional data are available from the corresponding authors on reasonable request.

## References

[1] Boyd, R. J., Avramopoulos, D., Jantzie, L. L. & McCallion, A. S. Neuroinflammation represents a common theme amongst genetic and environmental risk factors for Alzheimer and Parkinson diseases. *J. Neuroinflammation* **19**, 223 (2022). 36076238 10.1186/s12974-022-02584-x PMC9452283

[2] Zhang, W., Xiao, D., Mao, Q. & Xia, H. Role of neuroinflammation in neurodegeneration development. *Signal Transduct. Target Ther.* **8**, 267 (2023). 37433768 10.1038/s41392-023-01486-5 PMC10336149

[3] Colonna, M. & Butovsky, O. Microglia function in the central nervous system during health and neurodegeneration. *Annu Rev. Immunol.* **35**, 441–468 (2017). 28226226 10.1146/annurev-immunol-051116-052358 PMC8167938

[4] Tremblay, M. È et al. The role of microglia in the healthy brain. *J. Neurosci.* **31**, 16064–16069 (2011). 22072657 10.1523/JNEUROSCI.4158-11.2011 PMC6633221

[5] Arcuri, C., Mecca, C., Bianchi, R., Giambanco, I. & Donato, R. The pathophysiological role of microglia in dynamic surveillance, phagocytosis and structural remodeling of the developing CNS. *Front Mol. Neurosci.* **10**, 191 (2017). 28674485 10.3389/fnmol.2017.00191 PMC5474494

[6] Paolicelli, R. C. et al. Microglia states and nomenclature: a field at its crossroads. *Neuron* **110**, 3458–3483 (2022). 36327895 10.1016/j.neuron.2022.10.020 PMC9999291

[7] Davalos, D. et al. ATP mediates rapid microglial response to local brain injury in vivo. *Nat. Neurosci.* **8**, 752–758 (2005). 15895084 10.1038/nn1472

[8] Nimmerjahn, A., Kirchhoff, F. & Helmchen, F. Resting microglial cells are highly dynamic surveillants of brain parenchyma in vivo. *Science* **308**, 1314–1318 (2005). 15831717 10.1126/science.1110647

[9] Hickman, S., Izzy, S., Sen, P., Morsett, L. & El Khoury, J. Microglia in neurodegeneration. *Nat. Neurosci.* **21**, 1359–1369 (2018). 30258234 10.1038/s41593-018-0242-x PMC6817969

[10] Liu, T., Zhang, L., Joo, D. & Sun, S. C. NF-κB signaling in inflammation. *Signal Transduct. Target Ther.* **2**, 17023. 10.1038/sigtrans.2017.23 (2017). 29158945 10.1038/sigtrans.2017.23 PMC5661633

[11] Kofoed, R. H. & Aubert, I. Focused ultrasound gene delivery for the treatment of neurological disorders. *Trends Mol. Med.* **30**, 263–277 (2024). 38216449 10.1016/j.molmed.2023.12.006

[12] Meng, Y., Hynynen, K. & Lipsman, N. Applications of focused ultrasound in the brain: from thermoablation to drug delivery. *Nat. Rev. Neurol.* **17**, 7–22 (2021). 33106619 10.1038/s41582-020-00418-z

[13] Blackmore, J., Shrivastava, S., Sallet, J., Butler, C. R. & Cleveland, R. O. Ultrasound neuromodulation: a review of results, mechanisms and safety. *Ultrasound Med Biol.* **45**, 1509–1536 (2019). 31109842 10.1016/j.ultrasmedbio.2018.12.015 PMC6996285

[14] Yoo, S., Mittelstein, D. R., Hurt, R. C., Lacroix, J. & Shapiro, M. G. Focused ultrasound excites cortical neurons via mechanosensitive calcium accumulation and ion channel amplification. *Nat. Commun.* **13**, 493 (2022). 35078979 10.1038/s41467-022-28040-1 PMC8789820

[15] Darmani, G. et al. Non-invasive transcranial ultrasound stimulation for neuromodulation. *Clin. Neurophysiol.* **135**, 51–73 (2022). 35033772 10.1016/j.clinph.2021.12.010

[16] Beisteiner, R., Hallett, M. & Lozano, A. M. Ultrasound neuromodulation as a new brain therapy. *Adv. Sci.* **10**, e2205634 (2023). 10.1002/advs.202205634 PMC10190662 36961104

[17] Li, F. et al. Low-intensity pulsed ultrasound stimulation (LIPUS) modulates microglial activation following intracortical microelectrode implantation. *Nat. Commun.* **15**, 5512 (2024). 38951525 10.1038/s41467-024-49709-9 PMC11217463

[18] Grewal, S. et al. Using focused ultrasound to modulate microglial structure and function. *Front Cell Neurosci.* **17**, 1290628 (2023). 38164436 10.3389/fncel.2023.1290628 PMC10757935

[19] Chang, J. W., Wu, M. T., Song, W. S. & Yang, F. Y. Ultrasound stimulation suppresses LPS-induced proinflammatory responses by regulating NF-κB and CREB activation in microglial cells. *Cereb. Cortex* **30**, 4597–4606 (2020). 32248223 10.1093/cercor/bhaa062

[20] Li, D. et al. Low-intensity pulsed ultrasound dynamically modulates the migration of BV2 microglia. *Ultrasound Med. Biol.* **51**, 494–507 (2025). 39632209 10.1016/j.ultrasmedbio.2024.11.010

[21] Schregel, K. et al. Targeted blood brain barrier opening with focused ultrasound induces focal macrophage/microglial activation in experimental autoimmune encephalomyelitis. *Front Neurosci.* **15**, 665722 (2021). 34054415 10.3389/fnins.2021.665722 PMC8149750

[22] Lu, X. et al. Ultrasound stimulation modulates microglia M1/M2 polarization and affects hippocampal proteomic changes in a mouse model of Alzheimer’s disease. *Immun. Inflamm. Dis.* **12**, e70061 (2024). 39588954 10.1002/iid3.70061 PMC11590030

[23] Sung, C. Y., Chiang, P. K., Tsai, C. W. & Yang, F. Y. Low-intensity pulsed ultrasound enhances neurotrophic factors and alleviates neuroinflammation in a rat model of Parkinson’s disease. *Cereb. Cortex* **32**, 176–185 (2022). 10.1093/cercor/bhab201 34196669

[24] Hsu, C. H., Pan, Y. J., Zheng, Y. T., Lo, R. Y. & Yang, F. Y. Ultrasound reduces inflammation by modulating M1/M2 polarization of microglia through STAT1/STAT6/PPARγ signaling pathways. *CNS Neurosci. Ther.* **29**, 4113–4123 (2023). 37401041 10.1111/cns.14333 PMC10651950

[25] Murphy, K. R. et al. A practical guide to transcranial ultrasonic stimulation from the IFCN-endorsed ITRUSST consortium. *Clin. Neurophysiol.* **171**, 192–226 (2025). 39933226 10.1016/j.clinph.2025.01.004

[26] Aubry, J. F. et al. ITRUSST consensus on biophysical safety for transcranial ultrasound stimulation. *Brain Stimul.* **18**, 1896–1905 (2025). 41072763 10.1016/j.brs.2025.10.007 PMC12644226

[27] Zhao, J. et al. Neuroinflammation induced by lipopolysaccharide causes cognitive impairment in mice. *Sci. Rep.* **9**, 5790 (2019). 30962497 10.1038/s41598-019-42286-8 PMC6453933

[28] Martin, E. et al. Ultrasound system for precise neuromodulation of human deep brain circuits. *Nat. Commun.* **16**, 8024 (2025). 40913042 10.1038/s41467-025-63020-1 PMC12413462

[29] Brandi, E. et al. Brain region-specific microglial and astrocytic activation in response to systemic lipopolysaccharides exposure. *Front Aging Neurosci.* **14**, 910988 (2022). 36092814 10.3389/fnagi.2022.910988 PMC9459169

[30] Guo, Q. et al. NF-κB in biology and targeted therapy: new insights and translational implications. *Signal Transduct. Target Ther.* **9**, 53 (2024). 38433280 10.1038/s41392-024-01757-9 PMC10910037

[31] Kaltschmidt, B., Czaniera, N. J., Schulten, W. & Kaltschmidt, C. NF-κB in Alzheimer’s disease: friend or foe? Opposite functions in neurons and glial cells. *Int. J. Mol. Sci.* **25**, 11353 (2024). 39518906 10.3390/ijms252111353 PMC11545113

[32] Zong, B. et al. Mechanosensitive Piezo1 channel in physiology and pathophysiology of the central nervous system. *Ageing Res Rev.* **90**, 102026 (2023). 37532007 10.1016/j.arr.2023.102026

[33] Song, M., Zhang, M., He, S., Li, L. & Hu, H. Ultrasonic neuromodulation mediated by mechanosensitive ion channels: current and future. *Front Neurosci.* **17**, 1232308 (2023). 37583416 10.3389/fnins.2023.1232308 PMC10423872

[34] Jäntti, H. et al. Microglial amyloid beta clearance is driven by PIEZO1 channels. *J. Neuroinflammation* **19**, 147 (2022). 35706029 10.1186/s12974-022-02486-y PMC9199162

[35] Zhu, J. et al. The mechanosensitive ion channel Piezo1 contributes to ultrasound neuromodulation. *Proc. Natl. Acad. Sci. USA* **120**, e2300291120 (2023). 37098060 10.1073/pnas.2300291120 PMC10161134

[36] Menz, M. D. et al. Radiation force as a physical mechanism for ultrasonic neurostimulation of the ex vivo retina. *J. Neurosci.* **39**, 6251–6264 (2019). 31196935 10.1523/JNEUROSCI.2394-18.2019 PMC6687898

[37] Hertzberg, Y. et al. Ultrasound focusing using magnetic resonance acoustic radiation force imaging: application to ultrasound transcranial therapy. *Med. Phys.* **37**, 2934–2942 (2010). 20632605 10.1118/1.3395553

[38] Phipps, M. A. et al. Considerations for ultrasound exposure during transcranial MR acoustic radiation force imaging. *Sci. Rep.* **9**, 16235 (2019). 31700021 10.1038/s41598-019-52443-8 PMC6838326

[39] Ikai, H. et al. Low-intensity pulsed ultrasound accelerates periodontal wound healing after flap surgery. *J. Periodontal Res.* **43**, 212–216 (2008). 18302624 10.1111/j.1600-0765.2007.01016.x

[40] Seasons, G. M., Pellow, C., Kuipers, H. F. & Pike, G. B. Ultrasound and neuroinflammation: immune modulation via the heat shock response. *Theranostics* **14**, 3150–3177 (2024). 38855178 10.7150/thno.96270 PMC11155413

[41] Azadian, M. M. et al. Clearance of intracranial debris by ultrasound reduces inflammation and improves outcomes in hemorrhagic stroke models. *Nat Biotechnol*. 10.1038/s41587-025-02866-8 (2025). 10.1038/s41587-025-02866-8 PMC12951864 41214344

[42] Chen, T. T., Lan, T. H. & Yang, F. Y. Low-intensity pulsed ultrasound attenuates LPS-induced neuroinflammation and memory impairment by modulation of TLR4/NF-κB signaling and CREB/BDNF expression. *Cereb. Cortex* **29**, 1430–1438 (2019). 30873554 10.1093/cercor/bhy039

[43] Kim, J. et al. Repeated LPS induces training and tolerance of microglial responses across brain regions. *J. Neuroinflammation* **21**, 233 (2024). 39304952 10.1186/s12974-024-03198-1 PMC11414187

[44] Jung, H. et al. Differential regional vulnerability of the brain to mild neuroinflammation induced by systemic LPS treatment in mice. *J. Inflamm. Res* **15**, 3053–3063 (2022). 35645573 10.2147/JIR.S362006 PMC9140139

[45] Hasegawa-Ishii, S., Inaba, M. & Shimada, A. Widespread time-dependent changes in tissue cytokine concentrations in brain regions during the acute phase of endotoxemia in mice. *Neurotoxicology* **76**, 67–74 (2020). 31628962 10.1016/j.neuro.2019.10.006

[46] Masuda, T. et al. Spatial and temporal heterogeneity of mouse and human microglia at single-cell resolution. *Nature* **566**, 388–392 (2019). 30760929 10.1038/s41586-019-0924-x

[47] Tan, Y. L., Yuan, Y. & Tian, L. Microglial regional heterogeneity and its role in the brain. *Mol. Psychiatry* **25**, 351–367 (2020). 31772305 10.1038/s41380-019-0609-8 PMC6974435

[48] Tan, Z. et al. Morphological and functional differences between hippocampal and cortical microglia and its impact on neuronal over-excitation in a germline Pten mutant mouse model. *Neuroscience* **570**, 159–172 (2025). 39984030 10.1016/j.neuroscience.2025.02.044

[49] Valiukas, Z., Tangalakis, K., Apostolopoulos, V. & Feehan, J. Microglial activation states and their implications for Alzheimer’s Disease. *J. Prev. Alzheimers Dis.* **12**, 100013 (2025). 39800461 10.1016/j.tjpad.2024.100013 PMC12184064

[50] Müller, L. & Di Benedetto, S. Neuroimmune crosstalk in chronic neuroinflammation: microglial interactions and immune modulation. *Front Cell Neurosci.* **19**, 1575022 (2025). 40260075 10.3389/fncel.2025.1575022 PMC12009833

[51] Nakamura, A. & Shichita, T. Interaction between neurons and microglia in healthy and disease states. *Int. Immunol.*10.1093/intimm/dxaf057 (2025). 10.1093/intimm/dxaf057 41001810

[52] Zhong, M. Z., Peng, T., Duarte, M. L., Wang, M. & Cai, D. Updates on mouse models of Alzheimer’s disease. *Mol. Neurodegener.* **19**, 23 (2024). 38462606 10.1186/s13024-024-00712-0 PMC10926682

[53] Leinenga, G. & Götz, J. Scanning ultrasound removes amyloid-β and restores memory in an Alzheimer’s disease mouse model. *Sci. Transl. Med* .**7**, 278ra33 (2015). 10.1126/scitranslmed.aaa2512 25761889

[54] Leinenga, G. et al. Scanning ultrasound-mediated memory and functional improvements do not require amyloid-β reduction. *Mol. Psychiatry* **29**, 2408–2423 (2024). 38499653 10.1038/s41380-024-02509-5 PMC11412907

[55] Madore, C. et al. Early morphofunctional plasticity of microglia in response to acute lipopolysaccharide. *Brain, Behav., Immun.* **34**, 151–158 (2013). 23994463 10.1016/j.bbi.2013.08.008

[56] Zhang, A. Y. et al. Microglia reactivity is brain region and sex specific in the context of chronic stress. *Sci. Rep.* **15**, 33285 (2025). 41006452 10.1038/s41598-025-18000-2 PMC12475140

[57] Villa, A. et al. Sex-specific features of microglia from adult mice. *Cell Rep.* **23**, 3501–3511 (2018). 29924994 10.1016/j.celrep.2018.05.048 PMC6024879

[58] Fontana, F. et al. Development and validation of low-intensity pulsed ultrasound systems for highly controlled in vitro cell stimulation. *Ultrasonics* **116**, 106495 (2021). 34186322 10.1016/j.ultras.2021.106495

[59] Treeby, B. E. & Cox, B. T. k-Wave: MATLAB toolbox for the simulation and reconstruction of photoacoustic wave fields. *J. Biomed. Opt.* **15**, 021314 (2010). 20459236 10.1117/1.3360308

[60] Rosenhain, S. et al. A preclinical micro-computed tomography database including 3D whole body organ segmentations. *Sci. Data* **5**, 180294 (2018). 30561432 10.1038/sdata.2018.294 PMC6298256

[61] Livak, K. J. & Schmittgen, T. D. Analysis of relative gene expression data using real-time quantitative PCR and the 2(-Delta Delta C(T)) Method. *Methods* **25**, 402–408 (2001). 11846609 10.1006/meth.2001.1262

