# Supplementary material for: Ultrasound modulates microglial activity and reduces neuroinflammation in a parameter-dependent manner
Source: NPJ Acoust. 2026 May 6;2(1):15. doi: 10.1038/s44384-026-00047-8 (PMC13149327; doi:10.1038/s44384-026-00047-8)
Supplement: Supplementary file 1 — Supplemental Information [file 44384_2026_47_MOESM1_ESM.pdf]

Supplementary Material

Ultrasound modulates microglial activity and reduces neuroinflammation in a parameter-dependent manner

Authors

Sarina Grewal[1,2,3]*, Francesco Iacoponi[4,5], Lok Yin Nicholas Chan[1], Valeria Dosso[1], William Lim Kee Chang[1,6], Vanessa Drevenakova[1,3], Albert Ugwudike[7], Leonardo Ricotti[4,5], Paul M. Matthews[2,3,8], Andrea Cafarelli[4,5], Sophie V. Morse[1,3]*

*Corresponding authors:

s.grewal@imperial.ac.uk

sophie.morse11@imperial.ac.uk

Condition	Peak pressure (MPa)	Isppa (W/cm^2)	Ispta (W/cm^2)
In vitro	0.1	0.333	0.0667
In vitro	0.2	1.33	0.267
In vitro	0.4	5.33	1.07
In vivo (intensities after 2.7 dB skull attenuation)	0.2	0.707	0.141

Table S1. Calculated acoustic intensities for in vitro and in vivo experiments The spatial-peak pulse-average intensity (Isppa) and spatial-peak temporal-average intensity (Ispta) were derived for each tested pressure. These values are provided for comparison with previous literature.

Tissue type	Density (kg·m^{-3})	Speed of sound (m·s^{-1})	Absorption (db/(mhz^y·cm)	Power law expone nt (y)	Specific heat (j·kg^{-1}·k^{-1})	Thermal conductivity (w·m^{-1}·k^{-1})	Reference
Bone	1900	2242	2.7	1.18	1300	0.4	[1-3]
Tissue (water)	1000	1500	0.002	1.18	4180	0.6	[1]

Table S2. Acoustic and thermal properties assigned to bone (mouse skull) and soft tissue (water) in the simulations. Values include density, speed of sound, frequency-dependent absorption, power law exponent, specific heat capacity, and thermal conductivity.

Target gene	Forward primer (5′ to 3′)	Reverse primer (5′ to 3′)
TNF-α	CTGAACTTCGGGGTGATCGG	GGCTTGTCACTCGAATTTTGAGA
IL-1β	GCAACTGTTCCTGAACTCAACT	ATCTTTTGGGGTCCGTCAACT
IL-6	CTGCAAGAGACTTCCATCCAG	AGTGGTATAGACAGGTCTGTTGG
NF-κB	ATGGCAGACGATGATCCCTAC	CGGATCGAAATCCCCTCTGTT
IL-10	CTTACTGACTGGCATGAGGATCA	GCAGCTCTAGGAGCATGTGG
IL-4	GGTCTCAACCCCCAGCTAGT	GCCGATGATCTCTCTCAAGTGAT

GAPDH	CGACTTCAACAGCAACTCCCACTCTTC	TGGGTGGTCCAGGGTTTCTTACTCCTT

Table S3. List of primer sequences used for the qRT-PCR.

Parameter	Mouse tissue
Min microglia cell body diameter	3.86 mm
Microglia contrast threshold	0.6
Minimum microglia process optical density	0.32
Max microglia process radius	17.2 mm
Microglia max fragmentation length	5.44 mm
Activation process thickness	1.16 mm

Table S4. HALO microglial detection parameter summary.

Simulated acoustic pressure and temperature fields show negligible thermal effects in vivo

To estimate temperature changes induced by ultrasound in vivo, acoustic propagation was simulated using the k-Wave MATLAB toolbox, a k-space pseudospectral method-based solver [4]. Simulations concluded that peak temperature rises would remain below 0.02°C. The mean temperature increase observed in the skull was 0.0134 ± 0.0048 °C, while the regions surrounding the skull (i.e. brain and scalp) showed increases of 0.0117 ± 0.0038 °C. Figure S1 shows the temperature map in the coronal and sagittal planes across the brain, following 6 s of treatment for one targeted brain region. This suggests that thermal effects should be minimal under the selected in vivo parameter (0.5 MHz at 0.2 MPa). However, it should be noted that the model assumes homogeneous tissue properties and does not account for perfusion, thermoregulation, or nonlinear propagation. In addition, only a single treated region at a time

was simulated, and therefore cumulative thermal effects from repeated exposures were not evaluated.

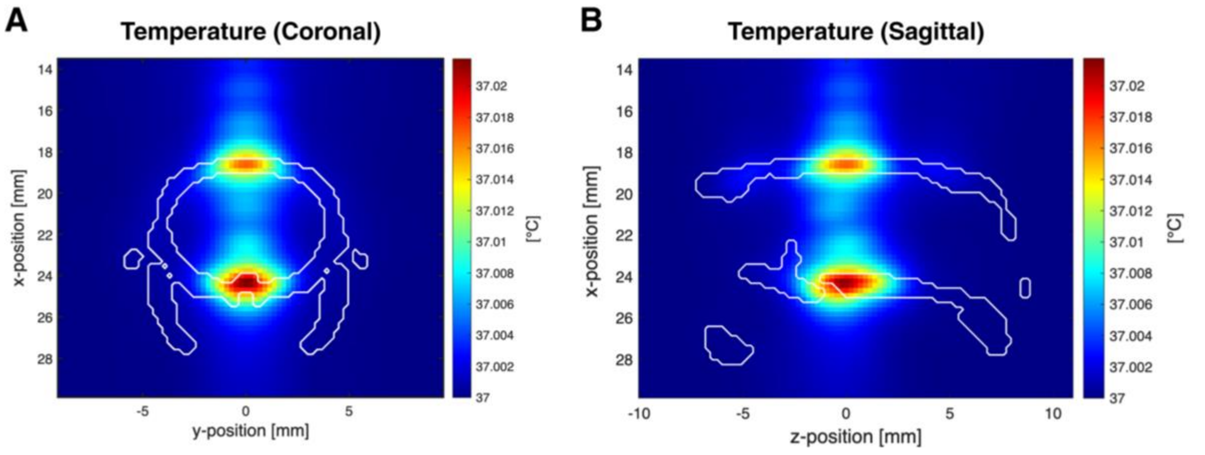

Fig S1. Simulated temperature field shows negligible thermal effects in vivo. The simulated temperature maps (A, B) illustrate that after 6 s of ultrasound, temperature increases remain below 0.02 °C, suggesting negligible heating. All maps are overlaid with a skull contour extracted from a CT-based anatomical model. A 0.5 MHz transducer emitting an acoustic pressure of 0.2 MPa was simulated in water using k-Wave's thermal solver from the calculated intensity and absorption parameters.

Supplementary Data

Data S1. Data supporting the findings of figures 1 – 6

References

1. Duck FA. Physical Properties of Tissues: A Comprehensive Reference Book. Oxford: Academic Press; 2013.
2. Estrada H, Rebling J, Turner J, Razansky D. Broadband acoustic properties of a murine skull. Phys Med Biol. 2016;61(5):1932-46. doi: 10.1088/0031-9155/61/5/1932.

3. Constans C, Mateo P, Tanter M, Aubry JF. Potential impact of thermal effects during ultrasonic neurostimulation: retrospective numerical estimation of temperature elevation in seven rodent setups. Phys Med Biol. 2018;63(2):025003. doi: 10.1088/1361-6560/aaa15c.
4. Treeby BE, Cox BT. k-Wave: MATLAB toolbox for the simulation and reconstruction of photoacoustic wave fields. J Biomed Opt. 2010;15(2):021314. doi: 10.1117/1.3360308.
